# Fluorescent Nanoparticle-RNAi-Mediated Silencing of Sterol Carrier Protein-2 Gene Expression Suppresses the Growth, Development, and Reproduction of *Helicoverpa armigera*

**DOI:** 10.3390/nano13020245

**Published:** 2023-01-06

**Authors:** Kexin Geng, Ying Zhang, Xi Zhao, Wanlin Zhang, Xinhan Guo, Lu He, Kaiyu Liu, Hong Yang, Huazhu Hong, Jianxin Peng, Rong Peng

**Affiliations:** Institute of Entomology, School of Life Sciences, Hubei Key Laboratory of Genetic Regulation and Integrative Biology, Central China Normal University, Wuhan 430079, China

**Keywords:** fluorescent nanoparticle, RNA interference, sterol carrier protein-2, *Helicoverpa armigera*, growth and development, pest control

## Abstract

*Helicoverpa armigera* is a polyphagous destructive lepidopteran pest with strong *Bacillus thuringiensis* (Bt) resistance. Cholesterol, a vital component for insect growth, can only be obtained from food, and its transfer and metabolism are regulated by sterol carrier protein-2 (SCP-2). This study examined whether *H. armigera SCP-2* (*HaSCP-2*) gene expression, involved in cholesterol absorption, can be silenced by nanocarrier fluorescent nanoparticle-RNA interference (FNP-RNAi) by larval feeding and whether the silencing affected *H. armigera* development. Fluorescence microscopy showed that nanoparticle-siRNA was distributed in Ha cells and the larval midgut. FNP-*HaSCP-2* siRNA suppressed *HaSCP-2* expression by 52.5% in *H.armigera* Ha cells. FNP can effectively help deliver siRNA into cells, protect siRNA, and is not affected by serum. FNP-siRNA *in vivo* biological assays showed that *HaSCP-2* transcript levels were inhibited by 70.19%, 68.16%, and 67.66% in 3rd, 4th, and 5th instar larvae, leading to a decrease in the cholesterol level in the larval and prepupal fatbodies. The pupation rate and adult emergence were reduced to 26.0% and 56.52%, respectively. This study demonstrated that FNP could deliver siRNA to cells and improve siRNA knockdown efficiency. *HaSCP-2* knockdown by FNP-siRNA *in vivo* hindered *H. armigera* growth and development. FNP could enhance RNAi efficiency to achieve pest control by *SCP-2*-targeted FNP-RNAi.

## 1. Introduction

The cotton bollworm *Helicoverpa armigera* (Lepidoptera: Noctuidae) is a widely distributed destructive lepidopteran agriculture pest that is polyphagous, highly adaptive, and has strong resistance [1,2,3]. More than 200 host plants are invaded and damaged by this pest in various ways, causing a huge decrease in crop yields and large global economic losses. The overuse of conventional pesticides not only has long-term negative impacts on the environment but also leads to strong pesticide resistance [2,3]. With the identification of insecticidal crystal toxins from the *Bacillus thuringiensis* (Bt), an aerobic, spore-forming, rod-shaped bacterium, transgenic Bt cotton was explored in pest control [4,5]. Moreover, with the large-scale cultivation of transgenic Bt cotton [4,5], the increasingly rapid evolution of Bt resistance poses a great threat to the application of Bt crops combating *H. armigera* [6,7]. Therefore, developing safe insecticides or effective methods with a new mode of action has drawn much attention in pest management.

Cholesterol is an essential nutrient for insect growth and development. It is part of the cellular membrane structure and an ecdysone precursor, which is a basic and critical substance for insect life activities [8,9,10]. Different from mammals, insects cannot synthesize cholesterol *de novo* because they lack the key enzymes required [11,12], and their normal growth and development depend on the uptake and transport of exogenous cholesterol or cholesterol analogs. The transport of exogenous cholesterol or cholesterol analogs depends on lipid transport proteins [13]. Sterol carrier protein-2 (SCP-2), a member of the SCP family belonging to non-specific lipid transfer proteins, plays an important role in intracellular cholesterol transport and metabolism in insects [14,15,16]. *Aedes aegypti* SCP-2 (AeSCP-2) was the first identified SCP-2 in insects, and it was shown to be involved in cholesterol uptake in mosquito larvae and was important for adult development and egg viability [17,18,19]. SCP-2 has also been identified in lepidopteran insects [20,21,22,23]. Midgut is the major site for insects to absorb cholesterol. The expression profiles of *SCP-2* showed that transcripts of *SCP-2* were detected strongly in the midgut during the larval stages, indicating that SCP-2 is involved in the cholesterol absorption in insects [17,20,21,22]. In *Bombyx mori*, cholesterol uptake significantly changed by overexpression or interference with *BmSCP-2* gene expression. Silencing of *BmSCP-2* gene expression resulted in the suppression of growth and development [20]. Similar results were also found in *Spodoptera litura* [21]. All these results suggest that SCP-2 plays a key role in cholesterol and lipid absorption and metabolism, which are critical for the growth and development of insects.

RNA interference (RNAi) was first reported in *Caenorhabditis elegans* [24]. This discovery led to the development of new therapies or pathways for human disease treatment, functional genomic research, and plant resistance to viruses [25]. In recent years, RNAi has shown great potential in exploring new insect pest management methods and developing late-model insecticides [26,27]. RNAi-based technology in insect pest control mainly comprises two approaches: targeted gene knockdown leading to insect death [28] and transgenic plants [29]. To meet the purpose of RNAi, different procedures can be selected such as microinjection, ingestion, soaking, and transfection [30]. Microinjection focuses on dsRNA delivery directly to the specific tissue and has high RNAi silencing efficiency. However, this method is mostly used in laboratory research. Because of high technical operation requirements, it cannot be applied to field pest control. Soaking and transfection experiments have been reported in studies with insect cell lines [31,32]. dsRNA-mediated RNAi by ingested dsRNA was assessed successfully in various species, such as *Tribolium castaneum* [33], *Epiphyas postvittana* [34], *Spodoptera exigua* [35], and *Blattella germanica* [36]. Ingestion of dsRNA in food is a high-throughput strategy for screening of target genes of insecticides, and is less labor-intensive, cost-effective, and simple. With these significant advantages, ingestion of siRNA by oral feeding shows great potential in field applications [37].

The effectiveness of RNAi mechanism mainly depends on the delivery, stability, and uptake of dsRNA by target pest species. Vectors are generally used to improve RNAi efficiency for insect pest control [37]. Current gene vectors are usually divided into three categories: liposomes [38,39], virus vectors [40], and synthetic vectors [41]. Synthetic vectors, such as nanoparticles, are rapidly updated and promising gene vectors because of their stability, ease of surface modification, and biodegradability or environmental safety. Recently, a cationic core–shell fluorescent nanoparticle (FNP) with a specially designed chemical structure was reported, which can be rapidly engulfed by live cells with low cytotoxicity yet high transfection efficacy [41]. The delicately designed FNP is composed of a fluorescent perylene-3,4,9,10-tetracarboxydiimide chromophore (PDI) center contributing to the visualizing of delivery process, a polyphenylene dendrimer-based inner layer to prevent PDI chromophore center from aggregation, and an outer cationic polymer shell for binding negatively charged nucleic acid. FNP can be an efficient gene carrier to silence target gene expression and genetically control pests [41].

In this study, to examine whether nanoparticle FNP-siRNA can inhibit *HaSCP-2* gene expression and subsequently suppress the growth and development of *H. armigera*, *HaSCP-2* was selected as the target gene, and FNP was employed as the gene carrier to orally deliver *HaSCP-2* siRNA into insects. The main objective was to lay a solid foundation for developing SCP-2-targeted nanopesticides and further exploring FNP-RNAi-based pest management methods.

## 2. Materials and Methods

### 2.1. Insects and Cells

*H. armigera* were purchased from Keyun Biopesticide Co., Ltd., Henan, China. Larvae were reared at 28 °C in 70–80% relative humidity with a light:dark photoperiod of 14 h:10 h. Larvae were fed an artificial diet in six-well plates till pupation. The artificial diet per liter contained 75 g soybean powder, 80 g wheat flour, 84 g maize powder, 20 g yeast extract (Sinopharm Chemical Reagent Co., Ltd., Shanghai, China), 2.5 g vitamin C (Chemstan Biotechnology Co., Ltd., Wuhan, China), 12.5 g sucrose (Sinopharm Chemical Reagent), 2 g sorbic acid (Sinopharm Chemical Reagent), 1.25 g nipagin (GuangCheng Chemical Reagent, Tianjin, China), and 12.5 g agar (Sinopharm Chemical Reagent). The moths were transferred to boxes covered with netted cloth and fed with 10% honey water.

The embryonic cell line QB-Ha-E5 (Ha cells) of *H. armigera* was obtained from Qingdao Agricultural University, Shandong, China [42]. The epidermal cell line of *H. armigera* (Ep cells) was gifted by Prof. Xiaofan Zhao [43] (Shandong University, Shandong, China) and stored in our laboratory. Cells were cultured in Grace’s insect medium (Gibco, Carlsbad, CA, USA) supplemented with 10% fetal bovine serum (Gibco) at 28 °C. Culture medium was refreshed every 2–3 days.

### 2.2. Detection of FNPs and siRNA in H. armigera Cells and Larval Midgut

Formation of FNP-siRNA complex: Nanocarrier FNP (5.6 ± 0.2 nm in Phosphate Buffered Saline) was prepared and kindly supplied by Prof. Jie Shen (Department of Entomology, China Agricultural University, Beijing, China) FAM-siRNA (siRNA labeled with a green fluorophore, FAM) was synthesized by GenePharma Co., Ltd., Shanghai, China (sequence is shown in Table 1). According to procedures provided by He et al. (2013) [41], for each transfection sample, we prepared FNP-siRNA complexes as follows: (a) 1 μg of siRNA was diluted in 50 μL Grace’s culture medium without serum and mixed gently; (b) 2 μL of FNP was diluted in 50 μL Grace’s culture medium without serum and incubated for 5 min at room temperature in the dark; (c) Then, combined the diluted siRNA and the diluted FNP, mixed gently, and incubated for 15 min at room temperature in the dark.

FNP and siRNA delivery: Ha cells were seeded in a 24-well cell culture plate at 2.5 × 10^5^ cells/well and were cultured overnight before transfection. The formed FNP-siRNA complexes were added to each well containing cells and medium and mixed gently_._ Incubate cells at 37 °C in a CO_2_ incubator for 18–48 h prior to fluorescence detection. The fluorescence of FNP and FAM-siRNA were detected and imaged using the EVOS FL Auto fluorescent imaging system (Life Technologies, Thermo Fisher Scientific, Waltham, MA, USA). To confirm that FNP and FAM-siRNA were delivered into the midgut of *H*. *armigera*, the 3rd instar larvae of *H*. *armigera* were fed an artificial diet of 5 µL of the FNP-FAM-siRNA complex containing 2.5 μg FAM-siRNA. A diet containing 5 μL DEPC-treated ddH_2_O was fed to *H*. *armigera* as the negative control. The midgut tissue from 4th instar larvae was dissected for fluorescence microscopy detection. Preparation of frozen sections of the midgut was as follows. The dissected midgut was embedded with optimum cutting temperature compound (OTC) and then quickly frozen in an acetone–dry ice bath. For sectioning, the samples were cut with a cryostat microtome and thaw-mounted on a glass slide. The slice thickness was 5–10 μm. The prepared frozen slices were then examined using the EVOS FL Auto fluorescent imaging system (Life Technologies).

### 2.3. Analysis of the Effect of Serum Presence on Cell Transfection Efficiency of FNP-siRNA

Ha and Ep cells were seeded in a 24-well cell culture plate, respectively. FNP and FAM-siRNA were pre-incubated at room temperature in the dark for 15 min in Grace’s culture medium containing 5% FBS or serum-free medium at FNP:siRNA amount ratios of 1:1, 2:1, and 3:1, respectively. Then, 100 μL of the FNP-siRNA complex was added to cells/well with 100 μL medium containing 5% FBS or serum-free medium, respectively. After 6–24 h transfection in darkness, fluorescence in FNP-siRNA transfected cells was imaged using the EVOS FL Auto fluorescent imaging system (Life Technologies). The transfection (delivery) efficiency was calculated as the percentage of fluorescent cells to total cells.

### 2.4. HaSCP-2 Expression Silencing in Ha Cells by FNP-siRNA

For RNAi, siRNA sequences that target *HaSCP-2* mRNA were designed (*HaSCP-2* siRNA sequence is shown in Table 1) and were synthesized by GenePharma (GenePharma Co., Ltd., Shanghai, China). Ha cells were seeded at a density of 70–80% in six-well cell culture plates, and cells attached to the bottom of the plate overnight. Three treatments were set as FNP-*HaSCP-2* siRNA-treated group (*HaSCP-2* siRNA + FNP group), *HaSCP-2* siRNA group, and wild type (WT; control group). The *HaSCP-2* siRNA + FNP group was treated with *HaSCP-2* siRNA (1 μg) and FNP (1 μL). The *HaSCP-2* siRNA group was treated with *HaSCP-2* siRNA (1 μg) and FuGENE HD Transfection Reagent (4 μL; Promega, Madison, WI, USA). qRT-PCR was performed to examine the *HaSCP-2* mRNA expression level in Ha cells at 24 h after FNP-*HaSCP-2* siRNA delivery.

### 2.5. FNP-HaSCP-2 siRNA Interference of the HaSCP-2 Gene by Oral Feeding in H. armigera

For siRNA oral feeding, ten 2nd instar larvae of *H. armigera* were synchronized for each experimental group. *HaSCP-2* siRNA was designed and synthesized by GenePharma (sequence is shown in Table 1). In the FNP-*HaSCP-2* siRNA-treated group, 2nd instar larvae were fed with 1 μg *HaSCP-2* siRNA and 2 μL FNP mixed in the artificial diet. The 2nd instar larvae were fed with 1 μg *HaSCP-2* siRNA and negative control (NC) siRNA, respectively, were used as the control. After food mixed with FNP-*HaSCP-2* siRNA was consumed in 5 h, larvae were transferred to the new plate containing fresh food without *HaSCP-2* siRNA and FNP. Then, larvae were reared to the 4th instar stage by feeding normal fresh food. Midgut and fatbody tissues from five randomly chosen 4th instar larvae were dissected. The *HaSCP-2* mRNA expression level in the midgut was determined by qRT-PCR. The fatbody was used for the cholesterol level assay in the following experiment.

### 2.6. qRT-PCR Analysis

Total RNA was extracted from Ha cells and *H*. *armigera* larvae using TRIzol reagent (Invitrogen, Carlsbad, CA, USA), respectively. Contaminant genomic DNA was removed from 2 μg total RNA samples using the TURBO DNA-free Kit (Ambion, Austin, TX, USA) according to the manufacturer’s instructions. The quantity of the RNA samples was determined via UV_260_ absorbance using the NanoDrop™ 1000 spectrophotometer (NanoDrop Products, Wilmington, DE, USA). First strand cDNA was reversely transcribed with 1 μg DNA-free total RNA using a High-Capacity cDNA Reverse Transcription Kit (Applied Biosystems™, Thermo Fisher Scientific, Waltham, MA, USA). A 25 ng first strand cDNA sample was used as the template for qRT-PCR with Fast EvaGreen^®^ qPCR Master Mix (Biotium, Fremont, CA, USA). The reaction included 7.5 μL of Fast EvaGreen^®^ qPCR Master Mix, 0.3 μL of forward primer, 0.3 μL of reverse primer, 8.2 μL of RNase-free water, and 25 ng of cDNA, in a total volume of 15 μL. qRT-PCR was performed using a CFX 96 Touch Real-Time PCR Detection System (Bio-Rad, Hercules, CA, USA) and the CFX manager software 3.0 (Bio-Rad). The amplification conditions were 3 min at 95 °C followed by 39 cycles of 10 s at 95 °C, 30 s at 60 °C, and 20 s at 72 °C. The reactions were set up in 96-well format microseal PCR plates (Bio-Rad) in triplicates. Transcript levels of *HaSCP-2* (GenBank JN582013) were normalized to the internal reference gene of ribosomal protein S3 (*rpS3A*) (GenBank XM_021337799.2) of *H*. *armigera*. Primers used for qRT-PCR analysis are listed in Table 1.

### 2.7. Cholesterol Level Assay

The larval fatbody was dissected in ice-cold phosphate-buffered saline (PBS, pH 7.4) from day 1 4th instar larvae. Tissues were put separately into 200 mL 1× lysis buffer (provided by Applygen Technologies Inc., Beijing, China) containing a protease inhibitor cocktail (Sigma-Aldrich, Saint Louis, MO, USA). Samples were homogenized with a micropestle and centrifuged at 2000× *g* for 5 min at 4 °C. The supernatant was snap-frozen in liquid nitrogen before storage at −80 °C. The cholesterol level of different samples was examined using the Tissue Total Cholesterol E1015 Assay Kit (Applygen Technologies Inc., Beijing, China) according to the manufacturer’s instructions. The protein concentration of the same sample was determined using the BCA Protein Assay Kit (Thermo Fisher Scientific Pierce, Rockford, IL, USA). A concentration standard curve (39–2500 µmol/L) of pure cholesterol (provided in the cholesterol assay kit) was constructed for each batch of assay, and the cholesterol amount in each sample was calculated using this cholesterol standard curve. Cholesterol concentration was defined as cholesterol unit/mg protein.

### 2.8. Biological Assays in H. armigera

Thirty-day 2 2nd instar larvae were combined to form a group and fed food mixed with FNP-*HaSCP-2* siRNA. For the FNP-*HaSCP-2* siRNA-treated group, 2nd instar larvae were fed with 1 μg *HaSCP-2* siRNA and 2 μL FNP mixed with the diet. Larvae fed with 1 μg NC siRNA and 2 μL FNP mixed in the diet formed the negative control group. The *HaSCP-2* siRNA group was treated with 1 μg *HaSCP-2* siRNA. WT larvae were fed with normal diet as the non-treated control group. Fresh food was provided every 1–2 days. Insect growth and development were monitored throughout the life cycle. Three independent replicates were performed for each experimental group. Larval body weight and length were measured daily, and the mortality was recorded. The number of eggs and offspring of each group were counted.

### 2.9. Statistical Analyses

Data were analyzed by analysis of variance (ANOVA) using the general linear model (GLM) procedure by GraphPad Prism software (v6.0; GraphPad Software, San Diego, CA, USA) to determine if the biological parameter in the treated and control groups significantly differed. Student’s *t*-test was used to determine the significance of differences when a pair of treatment groups with equal number of samples was compared.

## 3. Results

### 3.1. Fluorescence Images Visualizing the Distribution of FNP and siRNA in Ha Cells of H. armigera

**Figure 1:**
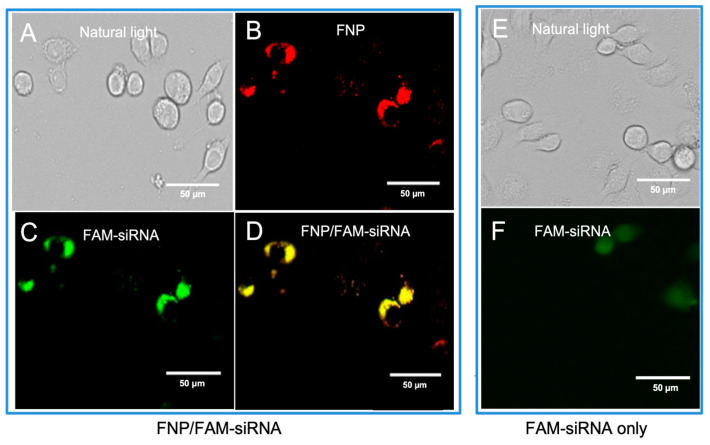
Fluorescence images of FNP–siRNA complexes in cells at 24 h after incubation (0.05 μM FNP, 100 μM DNA, N/P = 2:1, red: FNP, green: FAM fluorescence). For FNP/FAM-siRNA delivery group: (**A**) Bright-field channel. (**B**) Red fluorescence channel showing FNP. (**C**) Green fluorescence channel showing FAM-siRNA. (**D**) Fluorescence images of FNP-siRNA complexes ((**B**,**C**) merged). For the control group, FAM-siRNA only: (**E**) Bright-field channel. (**F**) Green fluorescence channel showing FAM-siRNA.

To determine the delivery of siRNA into *H. armigera* cells by fluorescent nanocarrier FNP, the FNP and FAM-siRNA (siRNA was labeled with the green fluorophore FAM) were mixed to form a complex and then added to Ha cells. The intracellular distribution of FNP and FAM-siRNA was examined by fluorescence microscopy 24 h post-transfection. siRNA transfection efficacy was determined by the fluorescent tracing of FNPs and FAM fluorophore-labeled siRNA in cells. FNP and FAM-siRNA were detected in the fluorescence images (Figure 1). Red fluorescence indicating FNP and green fluorescence indicating siRNA were localized to the cellular membrane and in cytoplasm. This demonstrated that FNPs could deliver exogenous siRNA in *H. armigera* Ha cells. This result was consistent with the previous study of FNP delivery in *Drosophila* S2 cells [41].

### 3.2. siRNA Delivery Efficiency of FNP In Vitro

**Figure 2:**
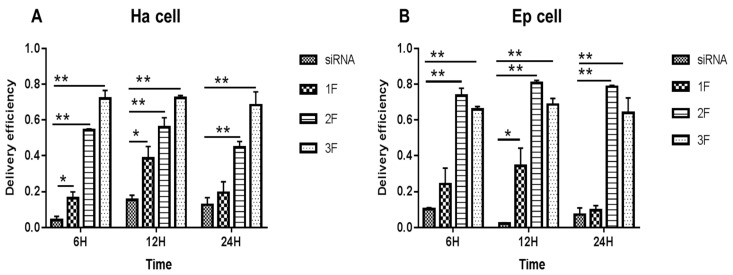
*In vitro* delivery efficiency of FNP–siRNA in two cell lines. (**A**) Delivery efficiency in *H. armigera* Ha cells. (**B**) Delivery efficiency in *H. armigera* Ep cells. 1F, 2F, and 3F indicate FNP:siRNA amount ratios 1:1, 2:1, and 3:1, respectively. Data are mean ± SD. Asterisks “**” and “*” indicate significance at *p* < 0.01 and *p* < 0.05 levels, respectively. After 6–24 h transfection in darkness, fluorescence in FNP-siRNA transfected cells was imaged. The delivery efficiency was calculated as the percentage of fluorescent cells to total cells.

In Ha and Ep cells (Figure 2), compared with the siRNA-treated group (no FNP added), the FNP-siRNA-treated group showed a significant increase in siRNA delivery efficiency at different times after incubation. The siRNA group showed a decreasing trend with incubation time or no large change of fluorescence; however, the FNP-siRNA-treated group showed an increasing trend of siRNA delivery efficiency with time. Thus, the nanocarrier FNP can effectively help deliver siRNA into cells and might protect siRNA from degradation. In Ha cells (Figure 2A), at 6 h after incubation of FNP and siRNA, the siRNA delivery efficiency of FNP in the FNP-siRNA-treated group increased by about 10.93% (*p* < 0.05), 49.93% (*p* < 0.01), and 67.93% (*p* < 0.01) at three FNP:siRNA ratios of 1:1, 2:1, and 3:1, compared with the siRNA group (no FNP added). At 12 h after incubation, the siRNA delivery efficiency of FNP increased by about 23.0% (*p* < 0.05), 43.49% (*p* < 0.01), and 58.0% (*p* < 0.01) at the three FNP:siRNA ratios above. At 24 h after incubation, the siRNA delivery efficiency of FNP in the FNP-siRNA-treated group increased by about 6.0%, 30.08% (*p* < 0.01), and 52.0% (*p* < 0.01). Similar results were found in Ep cells (Figure 2B). Compared with the siRNA group, the siRNA delivery efficiency of FNP in the FNP-siRNA-treated group increased by about 13.93%, 63.43% (*p* < 0.01), and 55.03% (*p* < 0.01) at 6 h after incubation of FNP and siRNA. At 12 h, siRNA delivery efficiency in the FNP-siRNA-treated group increased by about 32.09% (*p* < 0.05), 78.51% (*p* < 0.01), and 66.38% (*p* < 0.01). At 24 h, siRNA delivery efficiency in the FNP-siRNA-treated group increased by about 2.12%, 70.99% (*p* < 0.01), and 56.38% (*p* < 0.01). The experimental results of the Ep cell line were similar to the trend of the Ha cell line. Thus, with the increase in the FNP:siRNA ratio, more siRNAs were delivered into Ha cell, suggesting that increasing the FNP amount enhanced siRNA delivery efficiency in Ha cell line. While in Ep cells, the 2:1 ratio of FNP/siRNA is most effective. The result indicated that the ratio of FNP/siRNA with the highest delivery efficiency varied among different cell lines.

**Figure 3:**
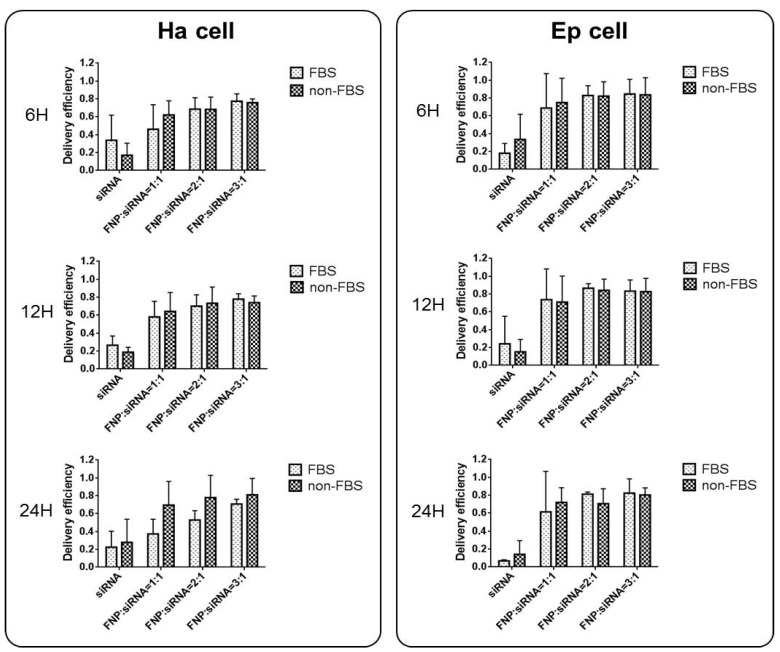
Effect of serum (FBS) on siRNA delivery efficiency in Ha and Ep cells. siRNA and FNP were incubated in a cell culture medium containing 5% FBS during siRNA delivery *in vitro*. For non-FBS cell groups, siRNA and FNP were incubated in a cell culture medium without FBS. siRNA delivery efficiency between FBS and non-FBS cell groups was not significantly different. The delivery efficiency was calculated as the percentage of fluorescent cells to total cells.

In addition, the effect of serum presence on siRNA delivery by FNP was examined. For FBS cell groups, siRNA and FNP were incubated in a cell culture medium containing 5% FBS during siRNA delivery *in vitro*. For non-FBS cell groups, siRNA and FNP were incubated in a cell culture medium without FBS. siRNA delivery efficiency between FBS and non-FBS cell groups was not significantly different (Figure 3). Therefore, FBS might not affect siRNA delivery efficiency if FNP is used as the gene carrier vector, which is different from liposome and other siRNA transfection reagents for RNAi *in vitro*. This discovery can simplify and facilitate RNAi experiments *in vitro* without the need for replacing the serum-free medium during siRNA transfection into cells.

### 3.3. Fluorescence Images Visualizing the Distribution of FNP and siRNA in the Midgut of H. armigera

**Figure 4:**
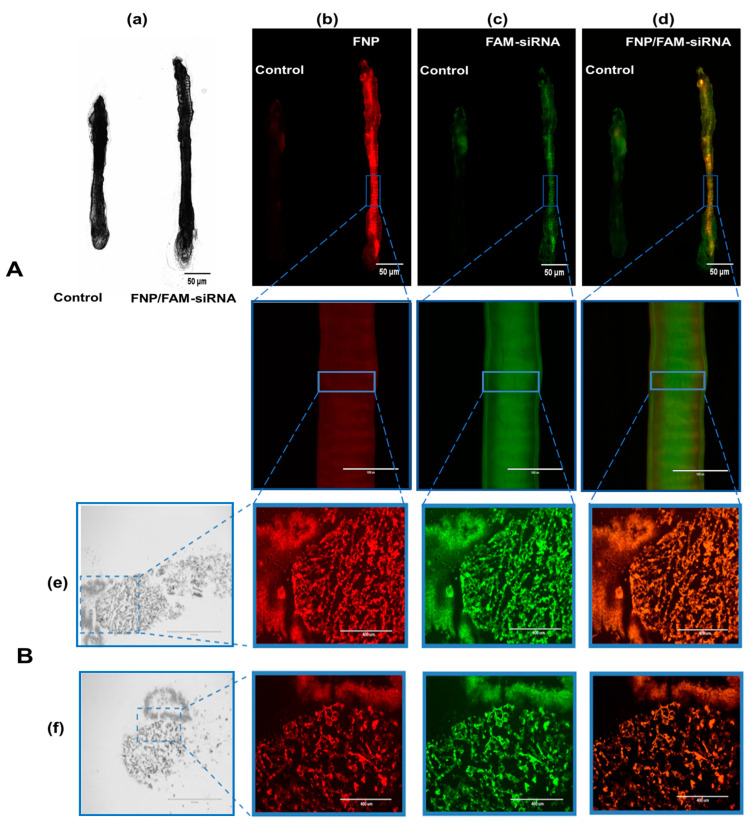
(**A**): Fluorescence images of dissected larval gut visualizing the distribution of FNP and siRNA. The 3rd instar larvae of *H*. *armigera* were fed an artificial diet of 5 µL of the FNP-FAM-siRNA complex containing 2.5 μg FAM-siRNA (red: FNP, green: FAM reference dye). A diet containing 5 μL DEPC-treated ddH_2_O was fed to *H*. *armigera* as the negative control. (**a**) Bright-field channel scanning. (**b**) Red fluorescence channel. (**c**) Green fluorescence channel. (**d**) Fluorescence of FNP–FAM-siRNA complexes. Scale bars (in left black-and-white micrographs and right upper three fluorescence micrographs) indicate 50 μm. Scale bars (in lower three of fluorescence micrographs) indicate 1000 μm. (**B**): Fluorescence images of FNP and siRNA in larval gut frozen sections. The mixture of FAM-siRNA and FNP was added to food to feed *H. armigera* larvae. After 24 h, the midgut was dissected and sectioned for observation. (**e**) Fluorescence images of the rip section. (**f**) Fluorescence images of the cross-section. FNP (red) and FAM-siRNA (green) were detected by fluorescence microscopy. Scale bars (in left black-and-white micrographs) indicate 1000 μm. Scale bars (in fluorescence micrographs) indicate 400 nm.

FAM-siRNA and FNP were visualized by two fluorescent channels, respectively. The control group presented weak autofluorescence or fluorescence contamination from food (Figure 4A). FNP and FAM-siRNA complexes were distributed in the midgut of *H. armigera*. To further confirm whether siRNA was delivered by FNP *in vivo*, frozen sections were prepared for detection. FNP and FAM-siRNA complexes were present in the midgut tissue (Figure 4B), suggesting that FNP-siRNA passed through the peritrophic membrane and entered midgut cells. Thus, FNP can act as a gene carrier to coat and deliver siRNA to suppress target gene expression in RNAi *in vivo*.

### 3.4. In Vitro HaSCP-2 Knockdown by FNP-siRNA in Ha Cells

**Figure 5:**
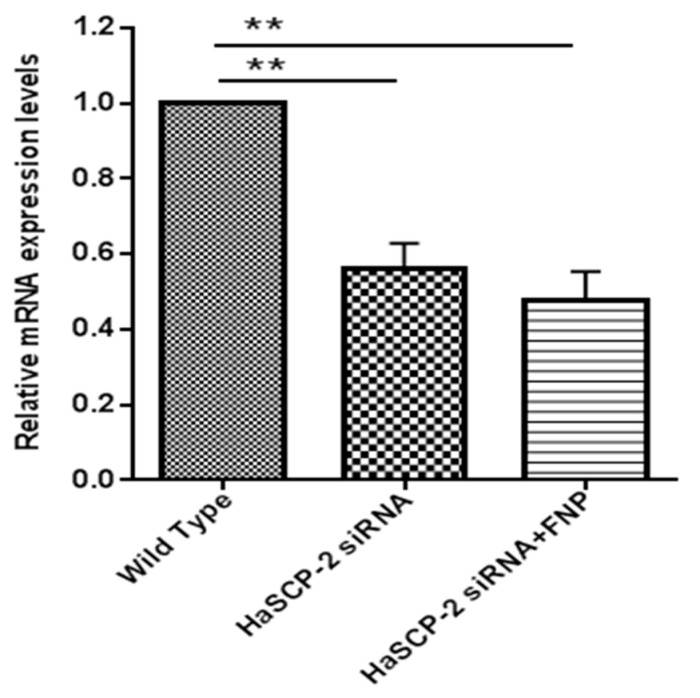
*HaSCP-2* mRNA expression level in Ha cells by FNP-RNAi *in vitro*. Three treatments were set as FNP-*HaSCP-2* siRNA-treated group (*HaSCP-2* siRNA + FNP group), *HaSCP-2* siRNA group, and wild type (WT; control group). Ha cells were treated with *HaSCP-2* siRNA and FNP (*HaSCP-2* siRNA + FNP group). The *HaSCP-2* siRNA group was treated with *HaSCP-2* siRNA and FuGENE HD transfection reagent. qRT-PCR was performed to examine the *HaSCP-2* mRNA expression level in Ha cells at 24 h after FNP-*HaSCP-2* siRNA delivery. Data are mean ± SD. Asterisk “**” indicates significance in the gene expression level between the treated and WT groups at *p* < 0.01 level (*t*-test).

*HaSCP-2* transcript levels decreased in the two *HaSCP-2* siRNA-treated groups. Compared with untreated WT cells, the *HaSCP-2* mRNA expression level in FNP-*HaSCP-2* siRNA-treated cells decreased by 52.5% (*p* < 0.01) (Figure 5). To compare the delivery effect of FNP with normal cell transfection reagent, another control was set for the *HaSCP-2* siRNA-treated group using FuGENE HD transfection reagent. *HaSCP-2* siRNA delivered by the FuGENE HD transfection reagent inhibited *HaSCP-2* mRNA expression by 44.11% (*p* < 0.01), lower than that of FNP-siRNA-treated cells (Figure 5). This illustrated that FNP, working as an apt *HaSCP-2* siRNA delivery vector, was an effective means of target gene knockdown *in vitro*.

### 3.5. In Vivo HaSCP-2 Knockdown by FNP-siRNA in H. armigera

**Figure 6:**
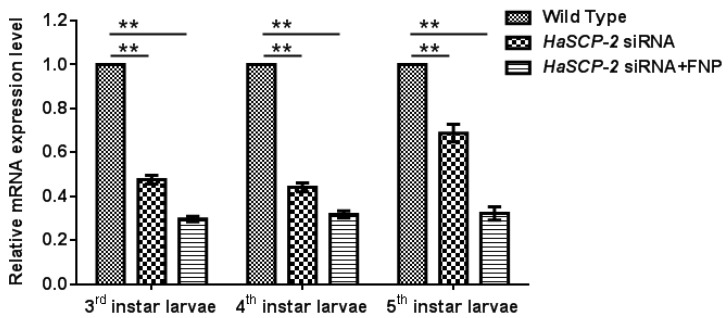
*HaSCP-2* expression level in the midgut of *H. armigera* by FNP-RNAi *in vivo*. Ten 2nd instar larvae were synchronized as a group and fed with FNP-*HaSCP-2* siRNA (*HaSCP-2* siRNA + FNP group). Midgut tissues from five individual larvae at 3rd, 4th, and 5th instar larvae, respectively, were collected, and the relative *HaSCP-2* mRNA expression levels in the larval midgut were determined by qRT-PCR. The 2nd instar larvae were fed with *HaSCP-2* siRNA and DEPC-treated ddH_2_O (Wild Type group), respectively, were used as the control. Data are mean ± SD. Asterisk “**” indicates significance at *p* < 0.01 level.

For *in vivo* FNP-RNAi study in *H. armigera*, ten 2nd instar larvae were combined to form a group and fed with FNP-*HaSCP-2* siRNA. Midgut tissues from five individual larvae at 3rd, 4th, and 5th instar larvae, respectively, were dissected, and the relative *HaSCP-2* mRNA expression levels in the larval midgut were determined by qRT-PCR. Thus, the relative *HaSCP-2* mRNA expression levels significantly reduced by about 70.19%, 68.16%, and 67.66% in the 3rd, 4th, and 5th instar larvae of the FNP-*HaSCP-2* siRNA-treated group (*p* < 0.05, Figure 6), compared with the untreated WT group. For the naked (without the FNP coating) *HaSCP-2* siRNA-treated group, the *HaSCP-2* transcript levels significantly decreased by about 52.34%, 55.81%, and 31.17% in the corresponding instar larvae (*p* < 0.05, Figure 6), which were lower than that of the FNP-HaSCP-2 siRNA-treated group.

The silencing effect by naked *HaSCP-2* siRNA faded with larval growth progression, which might be caused by siRNA degradation *in vivo*, whereas in FNP-*HaSCP-2* siRNA-treated larvae, the silencing effect by FNP-*HaSCP-2* siRNA showed no change in the three larval stages (Figure 6). This demonstrated that FNP coated *HaSCP-2* siRNA and protected it from degrading, indicating that FNP could enhance siRNA stability and improved its silencing function. For all stages, decline in the *HaSCP-2* expression level was much lower in the FNP-*HaSCP-2* siRNA-treated group than in the naked *HaSCP-2* siRNA-treated group, confirming that FNP could be used as a gene carrier to improve RNAi efficiency *in vivo*.

### 3.6. Effect of HaSCP-2 Knockdown on Cholesterol Accumulation by FNP-RNAi

**Figure 7:**
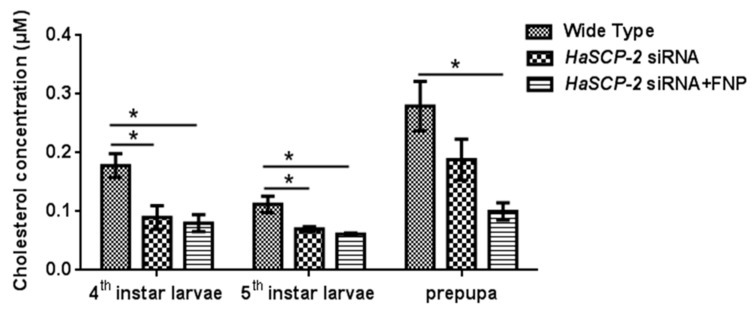
Cholesterol level in the fatbody. To study the effect of *HaSCP-2* knockdown on cholesterol accumulation by FNP-RNAi, ten 2nd instar larvae were synchronized as a group and fed with FNP-*HaSCP-2* siRNA (*HaSCP-2* siRNA + FNP group). Fatbody tissues from 3rd, 4th, and 5th instar larvae, respectively, were collected (five individuals/sample), and used for the cholesterol level assay. The 2nd instar larvae that fed with *HaSCP-2* siRNA and DEPC -treated ddH_2_O (Wild Type group), respectively, were set as the control. Data are mean ± SD. Asterisk “*” indicates significance at *p* < 0.05 level.

Compared with the WT group, the cholesterol level in the FNP-*HaSCP-2* siRNA-treated group reduced by about 55.05%, 45.38%, and 64.19% in the 4th and 5th instar and prepupal stages, respectively (*p* < 0.05, Figure 7). For the naked *HaSCP-2* siRNA-treated group, the cholesterol level reduced by about 49.58% (*p* < 0.05), 37.57% (*p* < 0.05), and 32.67% in the corresponding larval stages (Figure 7). The decrease in the cholesterol level was consistent with the decreasing trend of *HaSCP-2* gene expression. In insects, cholesterol was uptaken in the midgut and transported to fatbody for storage [44]. These results confirmed that HaSCP-2 was an important protein responsible for cholesterol transport in *H*. *armigera*. Therefore, the silencing of *HaSCP-2* expression by FNP-siRNA impaired the cholesterol uptake in the midgut, which led to the decline in cholesterol accumulation in the fatbody of *H*. *armigera*.

### 3.7. Effects of HaSCP-2 Silencing on the Growth, Development, and Reproduction of H. armigera by FNP-RNAi

**Figure 8:**
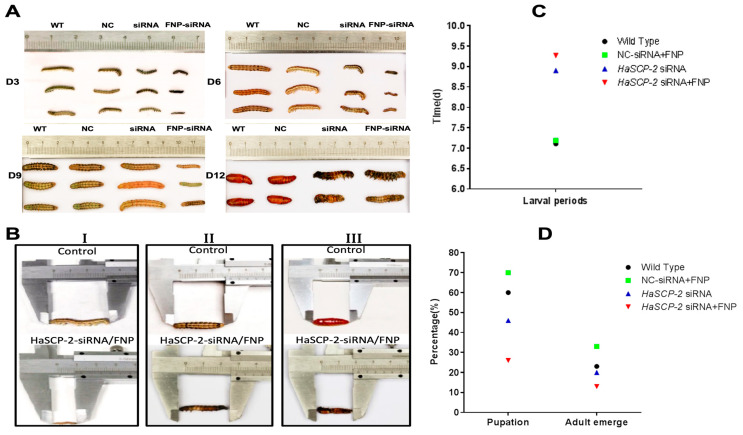
Effect of FNP-RNAi on growth and development. (**A**) 3, 6, 9, and 12 days after FNP-*HaSCP-2* siRNA oral feeding (WT: wild type; NC: FNP-negative control siRNA group; siRNA: *HaSCP-2* siRNA group; FNP-siRNA: FNP + *HaSCP-2* siRNA group). (**B**) Abnormal development of larvae and pupae resulted from FNP-*HaSCP-2* siRNA treatment ((**I**) Day 3 post siRNA ingestion; (**II**) Day 8 post siRNA ingestion; (**III**) Day 12 post siRNA ingestion). (**C**) Larval periods. (**D**) Pupation and adult emergence.

The FNP-*HaSCP-2* siRNA-treated group showed the smallest body size in the four experimental groups (Figure 8A). Three larvae from each group were randomly chosen as typical representatives to show the difference in body size between the WT and FNP-*HaSCP-2* siRNA group. In the FNP-*HaSCP-2* siRNA-treated group, pupation was delayed. (Figure 8A,B). Up to 12 days post feeding, for the FNP-*HaSCP-2* siRNA-treated group, the surviving larvae stayed in the 5th larval stage because of molting failure, whereas all WT larvae pupated normally (Figure 8A,B). Therefore, *HaSCP-2* siRNA carried by FNP interfered with *HaSCP-2* expression, resulting in the inhibition of cholesterol absorption in the larvae, which accordingly suppressed larval development. The FNP-NC siRNA-treated larvae showed no difference in larval growth compared with the WT, which eliminated the concern that FNP ingestion could lead to growth delay. Abnormal morphology of larvae and pupae is shown in Figure 8B. Compared with the normal control, FNP-*HaSCP-2* siRNA-treated 3rd instar larvae presented small body size (Figure 8(BI)), dark and stiff body in prepupae (Figure 8(BII)), and stagnant prepupa–pupa intermediates (Figure 8(BIII)). The life cycle of *H. armigera* in the four groups was monitored, respectively. The larval period of the FNP-*HaSCP-2* siRNA-treated group was approximately 9 days, showing a delay of 2–3 days compared with the 6 days of WT (Figure 8A,C). The pupation rate (Figure 8D) in the WT, FNP-NC siRNA-treated, and *HaSCP-2* siRNA-treated groups was about 60%, 70%, and 46%, respectively; however, the pupation rate in the FNP-*HaSCP-2* siRNA-treated group decreased to 26.0%. Subsequently, the emergence rate (Figure 8D) in the FNP-*HaSCP-2* siRNA-treated group decreased to 13.0%, whereas that of the WT, FNP-NC siRNA-treated, and *HaSCP-2* siRNA-treated groups was 23.0%, 33.0%, and 20.0%.

**Figure 9:**
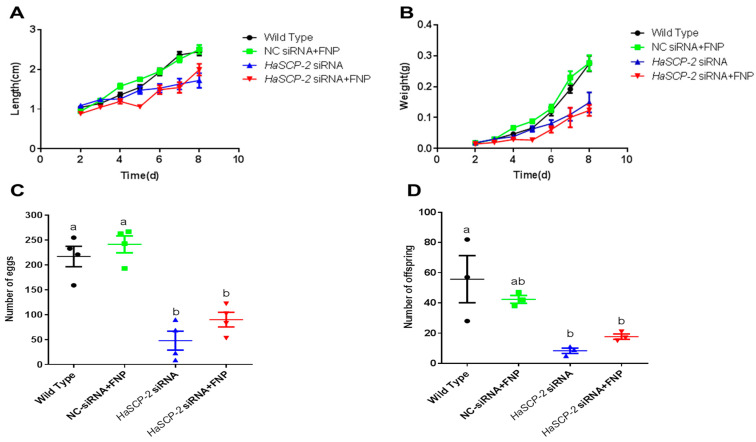
Life cycle analyses. (**A**) Analysis of average larval body length from day 2 to 8 post RNAi. (**B**) Analysis of average larval body weight from day 2 to 8 post RNAi. (**C**) Number of eggs. (**D**) Number of offspring. Data are mean ± SD. Different letters above bars indicate that the mean values differ from each treatment significantly (*p* < 0.05, Tukey’s HSD multiple test) between the different treatments.

The FNP-*HaSCP-2* siRNA-treated group showed lower body weight and length than that of WT control group and NC-siRNA/FNP group (Figure 9). Periodic tracking of the body weight and length was performed for all surviving samples. The larvae in FNP-*HaSCP-2* siRNA-treated group grew slower than those in the control group from the day 1 3rd instar stage to the day 3 5th instar stage (Figure 9A,B). The number of eggs or offspring in the FNP-*HaSCP-2* siRNA-treated group was about 33.0% of that in the WT group (Figure 9C,D), indicating that the lack of SCP-2 blocked the cholesterol storage required to form new tissues or organs during the pupal stage, causing higher mortality and less fecundity of the population. Thus, *HaSCP-2* knockdown by FNP-RNAi hindered the growth and development of *H. armigera*.

## 4. Discussion

The RNAi-based pest control strategy needs a suitable dsRNA delivery system to ensure high insecticidal efficiency and simple field application. In a previous study, a cationic core–shell FNP was used as vector for delivering dsRNA to silence *CHT-10* gene expression in corn borer larvae [41]. Fluorescent core–shell nanoparticles have the advantages of high gene delivery efficiency, low cytotoxicity, traceable fluorescence, and easy operation *in vivo*. Therefore, we employed the nanocarrier FNP to silence the *HaSCP-2* gene expression. In this study, the fluorescence images showed the distribution of FNP–siRNA in *H. armigera* cultured cells and midgut tissue, confirming that FNP met the gene carrier requirement of delivering exogenous siRNA into gut cells by oral feeding. qRT-PCR demonstrated that the ingestion of FNP-*HaSCP-2* siRNA successfully knocked down the target gene, and FNP could enhance siRNA stability and improve RNAi efficiency *in vivo*.

In this study, two cell lines were used to study the effects of different FNP:siRNA mixing ratios on siRNA delivery efficiency. The amount of FNP could affect siRNA delivery efficiency. When the FNP:siRNA ratios were 2:1 and 3:1, the efficiency significantly increased. The optimal FNP/siRNA ratio in Ha cells is 3:1, while that in Ep cells is 2:1. The difference demonstrated that the optimal amount of FNP used in this method varied in different cell lines, and high amount of FNP was not necessary for highly efficient delivery in Ep cells. This result suggested that low amount of FNP could probably achieve a good delivery efficiency in some cells. In addition, the presence or absence of serum had no effect on siRNA delivery efficiency. This fact was different from conventional liposome transfection reagents, which were negatively affected by serum. The drawback of cationic liposome is that unknown complicated protein components in serum enter cells during transfection, causing cytotoxicity and resulting in the failure of transfection. The FNP nanocarrier could only effectively enclose negative charged nuclear acid without carrying serum protein into cells. Moreover, in FNP transfection, a certain amount of antibiotics can be used for cell culture to avoid microorganism contamination and promote transfection efficiency. Thus, FNP has more advantages compared with liposome transfection reagents in the prospective of serum influence, operation simplification, and transfection efficiency improvement. Moreover, the fluorescence of FAM-siRNA and FNP increased with time, indicating that FNP could protect siRNA from degrading, and the FNP–siRNA complex enhanced the effect of RNAi *in vitro*.

Although chemical pesticide application is the main method of pest control, it has caused severe environmental pollution and insect resistance. In RNAi technology development, green synthetic carriers came into being to protect the environment. Chitosan nanoparticles, fluorescent cationic dendrimer generation 2, carbon quantum dots, silica, perylene diimide-cored poly amino acids, and a formulation of the nanocarrier/dsRNA/detergent have been used to target different genes [45,46,47,48]. They could penetrate the physiological defense of insects in different ways and improved RNAi efficiency. The nanoparticle-mediated delivery system could be optimized according to the research status of different insects to explore integrated pest management methods. This study provides a comparative analysis of a nanoparticle-RNAi delivery system for gene knockdown and potential RNAi-based bioinsecticide investigation. HaSCP-2 structure analysis by NMR showed that HaSCP-2 acted as a carrier for cholesterol and lipids, which facilitated the screening of effective insecticides targeting insect cholesterol metabolism [23]. The *HaSCP-2* expression level was reduced by 67.66% in 5th instar larvae compared with that of the wild type. This is similar to the knockdown of *HaSCP-2* gene by dsRNA microinjection in 5th larva of *H. armigera* [22]. Our advantage lies in the employment of the advanced nanocarrier FNP to deliver siRNA into *H. armigera* through an easy-operation approach, oral feeding, which resulting in the same knockdown effect *in vivo* as that of the microinjection delivery method. Consequently, the cholesterol level in the fatbody decreased by 64.19%. This further confirmed that *HaSCP-2* was a critical gene for cholesterol uptake and transport *in vivo* and that the HaSCP-2 silencing may lead to cholesterol uptake reduction, lipid mobilization and metabolism impairment. Hence, delayed growth in life cycle stages, reduced body weight and length, malformation and mortality, and drastically reduced offspring number were observed in bioassays. These results indicated that *HaSCP-2* was important for the growth and development of *H. armigera* and could be a potential target for pest control. Therefore, according to the findings in this study, the *HaSCP-2* gene can be an ideal target for RNAi-based pest control in the development of green and efficient pest management strategies. This FNP-RNAi-based pest management method shows great potential in agriculture applications. More in-depth research on nanomaterials delivery systems needs to be performed in the future in minimizing the environmental impact of agrochemicals [49,50]. Additionally, one of the factors that limit the feasibility of this approach is the cost in terms of FNP-siRNA complex, which needs to be improved in future. We could combine this approach with pesticides or biological methods to realize environmentally friendly pest management and sustainable agriculture development.

## Figures and Tables

**Table 1:** Primers for quantitative real-time PCR and siRNA.

Primer Name	Sequence (5′→3′)	Length (Base)	Tm	Efficiency
Q HaSCP-2F	GAGAAAGTACGAGGCATCTATGG	23	58	99%
Q HaSCP-2R	ATCGCTGATGGTGAAGGTG	19	55	
Q rpS3A-F	CCTCGTGAGGCCAACAT	17	54	98%
Q rpS3A-R	TGATGGCACGGACCTGAGT	19	57	
HaSCP-2 siRNA	Sense:5′-GCAAAUGAGCUGAUCACAUTT-3′ Antisense: 5′-AUGUGAUCAGCUCAUUUGCTT-3′	21		
FAM-siRNA (Negative control)	Sense:5′-UUCUCCGAACGUGUCACGUTT-3′ Antisense: 5′-ACGUGACACGUUCGGAGAATT-3′	21		

## Data Availability

Not applicable.

## References

[1] Cunningham J., Zalucki M., West S. (1999). Learning in *Helicoverpa armigera* (Lepidoptera: Noctuidae): A new look at the behaviour and control of a polyphagous pest. Bull. Entomol. Res..

[2] Brcbvault T., Oumarou Y., Achaleke J., Vaissayre M., Nibouche S. (2009). Initial activity and persistence of insecticides for the control of bollworms (Lepidoptera: Noctuidae) in cotton crops. Crop Prot..

[3] Joseph A., Thierry B. (2010). Inheritance and stability of pyrethroid resistance in the cotton bollworm *Helicoverpa armigera* (Lepidoptera: Noctuidae) in Central Africa. Pest Manag Sci..

[4] Gassmann A., Carrière Y., Tabashnik B. (2009). Fitness Costs of Insect Resistance to *Bacillus thuringiensis*. Annu. Rev. Entomol..

[5] Li G., Wu K., Gould F., Wang J., Miao J., Gao X., Guo Y. (2007). Increasing tolerance to Cry1Ac cotton from cotton bollworm, *Helicoverpa armigera*, was confirmed in Bt cotton farming area of China. Ecol. Entomol..

[6] Jin L., Zhang H., Lu Y., Yang Y., Wu K., Tabashnik B., Wu Y. (2015). Large-scale test of the natural refuge strategy for delaying insect resistance to transgenic Bt crops. Nat. Biotechnol..

[7] Jin L., Wang J., Guan F., Zhang J., Yu S., Liu S., Xue Y., Li L., Wu S., Wang X. (2018). Dominant point mutation in a tetraspanin gene associated with field-evolved resistance of cotton bollworm to transgenic Bt cotton. Proc. Natl. Acad. Sci. USA.

[8] Goldstein J., Brown M. (1990). Regulation of the mevalonate pathway. Nature.

[9] Lasser N., Clayton R. (1966). The intracellular distribution of sterols in *Eurycotis floridana* and its possible relation to subcellular membrane structures. J. Lipid Res..

[10] Borovsky D., Whisenton L., Thomas B., Morton S. (1986). Biosynthesis and distribution of ecdysone and 20-OH-ecdysone in *Aedes aegypti*. Arch Insect Biochem..

[11] Jouni Z., Zamora J., Wells M. (2002). Absorption and tissue distribution of cholesterol in *Manduca sexta*. Arch Insect Biochem..

[12] Nes W., Lopez M., Zhou W., Guo D., Dowd P., Norton R. (1997). Sterol utilization and metabolism by *Heliothis zea*. Lipids.

[13] Jing X., Behmer S. (2020). Insect sterol nutrition: Physiological mechanisms, ecology, and applications. Annu. Rev. Entomol..

[14] Vyazunova I., Lan Q., Liu N. (2008). Insect sterol carrier protein-2 gene family: Structures and functions. Recent Adv. Insect Physiol. Toxicol. Mol. Biol..

[15] Lan Q., Massey R. (2004). Subcellular localization of the mosquito sterol carrier protein-2 and sterol carrier protein-x. J. Lipid Res..

[16] Kim M., Wessely V., Lan Q. (2005). Identification of mosquito sterol carrier protein-2 inhibitors. J. Lipid Res..

[17] Krebs K., Lan Q. (2003). Isolation and expression of a sterol carrier protein-2 gene from the yellow fever mosquito, *Aedes aegypti*. Insect Mol. Biol..

[18] Radek J., Dyer D., Lan Q. (2010). Effects of mutations in *Aedes aegypti* sterol carrier protein-2 on the biological function of the protein. Biochemistry..

[19] Peng R., Fu Q., Hong H., Tyler S., Lan Q. (2017). THAP and ATF-2 regulated sterol carrier protein-2 promoter activities in the larval midgut of the yellow fever mosquito, *Aedes aegypti*. PLoS ONE.

[20] Gong J., Hong Y., Zha X., Lu C., Zhu Y., Xia Q. (2006). Molecular cloning and characterization of *Bombyx mori* sterol carrier protein x/sterol carrier protein 2 (SCPx/SCP2) gene. Mitochondrial DNA.

[21] Guo X., Zheng S., Liu L., Feng Q. (2009). The sterol carrier protein 2/3-oxoacyl-CoA thiolase (SCPx) is involved in cholesterol uptake in the midgut of *Spodoptera litura*: Gene cloning, expression, localization and functional analyses. BMC Mol. Biol..

[22] Du X., Ma H., Zhang X., Liu K., Peng J., Lan Q. (2012). Characterization of the sterol carrier protein-x/sterol carrier protein-2 gene in the cotton bollworm, *Helicoverpa armigera*. J. Insect Physiol..

[23] Ma H., Ma Y., Liu X., Dyer D., Xu P., Liu K. (2015). NMR structure and function of *Helicoverpa armigera* sterol carrier protein-2, an important insecticidal target from the cotton bollworm. Sci Rep..

[24] Fire A., Xu S., Montgomery M., Kostas S., Driver S., Mello C. (1998). Potent and specific genetic interference by double-stranded RNA in *Caenorhabditis elegans*. Nature.

[25] Zamore P. (2001). RNA interference: Listening to the sound of silence. Nat. Struct Biol..

[26] Zhang H., Li H., Miao X. (2013). Feasibility, limitation and possible solutions of RNAi-based technology for insect pest control. Insect Sci..

[27] Huvenne H., Smagghe G. (2009). Mechanisms of dsRNA uptake in insects and potential of RNAi for pest control: A review. J. Insect Physiol..

[28] Bettencourt R., Terenius O., Faye I. (2002). Hemolin gene silencing by ds-RNA injected into Cecropia pupae is lethal to next generation embryos. Insect Mol. Biol..

[29] Mao Y., Cai W., Wang J., Hong G., Tao X., Wang L. (2007). Silencing a cotton bollworm P450 monooxygenase gene by plant-mediated RNAi impairs larval tolerance of gossypol. Nat. Biotechnol..

[30] Yu N., Christiaens O., Liu J., Niu J., Cappelle K., Caccia S. (2013). Delivery of dsRNA for RNAi in insects: An overview and future directions. Insect Sci..

[31] Clemens J., Worby C., Simonson L., Muda M., Maehama T., Hemmings B. (2000). Use of double-stranded RNA interference in *Drosophila* cell lines to dissect signal transduction pathways. Proc. Natl. Acad. Sci. USA.

[32] Caplen N., Fleenor J., Fire A., Morgan R. (2000). dsRNA-mediated gene silencing in cultured *Drosophila* cells: A tissue culture model for the analysis of RNA interference. Gene.

[33] Wang J., Wu M., Wang B., Han Z. (2013). Comparison of the RNA interference effects triggered by dsRNA and siRNA in *Tribolium castaneum*. Pest Manag. Sci..

[34] Turner C., Davy M., MacDiarmid R., Plummer K., Birch N., Newcomb R. (2006). RNA interference in the light brown apple moth, *Epiphyas postvittana* (Walker) induced by double-stranded RNA feeding. Insect Mol. Biol..

[35] Surakasi V., Mohamed A., Kim Y. (2011). RNA interference of β1 integrin subunit impairs development and immune responses of the beet armyworm, *Spodoptera exigua*. J. Insect Physiol..

[36] Lin Y., Huang J., Liu Y., Belles X., Lee H. (2017). Oral delivery of dsRNA lipoplexes to German cockroach protects dsRNA from degradation and induces RNAi response. Pest Manag. Sci..

[37] Joga M., Zotti M., Guy S. (2016). RNAi efficiency, systemic properties, and novel delivery methods for pest insect control: What We Know So Far. Front. Physiol..

[38] Rooijen N., Nieuwmegen R. (2009). Liposomes in immunology: Multilamellar phosphatidylcholine liposomes as a simple, biodegradable and harmless adjuvant without any immunogenic activity of its own. Immunol. Commun..

[39] Taning C., Christiaens O., Berkvens N., Casteels H., Maes M., Smagghe G. (2016). Oral RNAi to control *Drosophila suzukii*: Laboratory testing against larval and adult stages. J. Pest Sci..

[40] Khan A., Ashfaq M., Kiss Z., Khan A., Mansoor S., Falk B. (2017). Use of recombinant tobacco mosaic virus to achieve RNA interference in plants against the citrus mealybug, *Planococcus citri* (Hemiptera: Pseudococcidae). PLoS ONE.

[41] He B., Chu Y., Yin M., Müllen K., An C., Shen J. (2013). Fluorescent nanoparticle delivered dsRNA toward genetic control of insect pests. Adv. Mater..

[42] Zheng G., Li C., Zhou H., Li S., Li G. (2010). Establishment of two new cell lines from the embryonic tissue of *Helicoverpa armigera* (Lepidoptera: Noctuidae) and their responses to baculovirus infection. Acta Entomol. Sin..

[43] Shao H., Zheng W., Liu P., Wang Q., Wang J., Zhao X. (2008). Establishment of a new cell line from lepidopteran epidermis and hormonal regulation on the genes. PLoS ONE.

[44] Kuthiala A., Ritter K. (1988). Esterification of cholesterol and cholestanol in the whole body, tissues, and frass of *Heliothis zea*. Arch Insect Biochem. Physiol..

[45] Zhang X., Zhang J., Zhu K. (2010). Chitosan/double-stranded RNA nanoparticle-mediated RNA interference to silence chitin synthase genes through larval feeding in the African malaria mosquito (*Anopheles gambiae*). Insect Mol. Biol..

[46] Shen D., Zhou F., Xu Z., He B., Li M., Shen J. (2014). Systemically interfering with immune response by a fluorescent cationic dendrimer delivered gene suppression. J. Mater. Chem. B.

[47] Yang Y., Jiang Q., Peng M., Zhou Z., Du X., Yin M., Shen J., Yan S. (2022). A star polyamine-based nanocarrier delivery system for enhanced avermectin contact and stomach toxicity against green peach aphids. Nanomaterials.

[48] Das S., Debnath N., Cui Y., Unrine J., Palli S. (2015). Chitosan carbon quantum dot, and silica nanoparticle mediated dsRNA delivery for gene silencing in *Aedes aegypti*: A comparative analysis. ACS Appl. Mater. Interfaces.

[49] Chariou P., Ortega-Rivera O., Steinmetz N. (2020). Nanocarriers for the delivery of medical, veterinary, and agricultural active ingredients. ACS Nano..

[50] Yan S., Li N., Guo Y., Chen Y., Ji C., Yin M., Shen J., Zhang J. (2022). Chronic exposure to the star polycation (SPc) nanocarrier in the larval stage adversely impairs life history traits in *Drosophila melanogaster*. J. Nanobiotechnol..

